# The backpack quotient filter: A dynamic and space-efficient data structure for querying *k*-mers with abundance

**DOI:** 10.1016/j.isci.2024.111435

**Published:** 2024-11-23

**Authors:** Victor Levallois, Francesco Andreace, Bertrand Le Gal, Yoann Dufresne, Pierre Peterlongo

**Affiliations:** 1 University Rennes, Inria, CNRS, IRISA - UMR 6074, 35000 Rennes, France; 2 Department of Computational Biology, Institut Pasteur, Université Paris Cité, 75015 Paris, France; 3 Bioinformatics and Biostatistics Hub, Institut Pasteur, Université de Paris, 75015 Paris, France; 4 University Rennes, Inria, CNRS, IRISA - Taran team, ENSSAT, Lannion, France; 5 Sorbonne Université, Collège doctoral, 75005 Paris, France

**Keywords:** Biocomputational method, Genomic analysis, High-performance computing in bioinformatics

## Abstract

Genomic data sequencing is crucial for understanding biological systems. As genomic databases like the European Nucleotide Archive expand exponentially, efficient data manipulation is essential. A key challenge is querying these databases to determine the presence or absence of specific sequences and their abundance within datasets.

This paper presents the Backpack Quotient Filter (BQF), a data structure for indexing *k*-mers (substrings of length *k*), which offers greater space efficiency than the Counting Quotient Filter (CQF). The BQF maintains essential features such as abundance information and dynamicity, with an extremely low false positive rate of less than $10^{-5}\%$. Our method redefines abundance information handling and implements an independent strategy for space efficiency.

The BQF uses four times less space than the CQF on complex datasets such as sea-water metagenomics sequences. Additionally, its space efficiency improves with larger datasets, addressing the need for scalable data solutions.

## Highlights

• The BQF is 3–5 times more space efficient than its previous alternative
• The BQF is inexact but experiments show a 10^−5^% false positive rate for queries
• The more data we index, the more space-efficient (bits/element) the BQF is
• It is well-suited for metagenomics, with complex and low-redundancy data

## Graphical abstract

**Figure:**
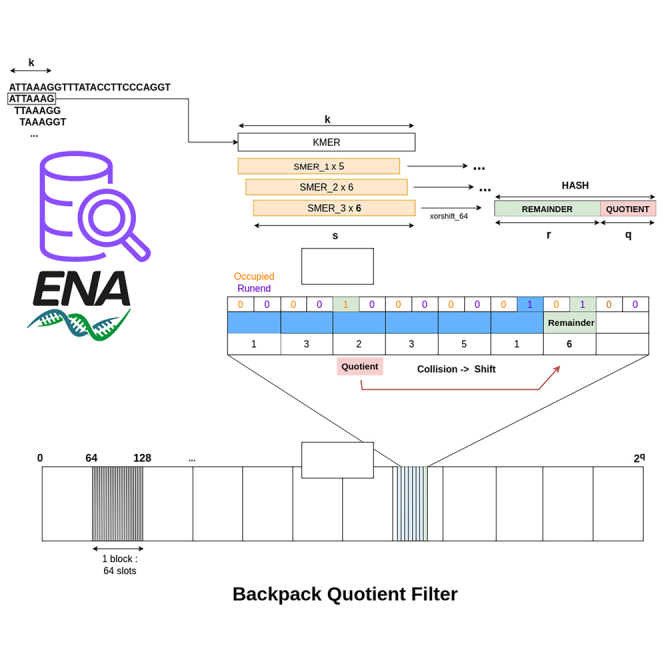

## Introduction

Genomic data sequencing is a powerful tool for understanding the intricacies of biological systems. Sequencing produces plain text, organized as reads in files. Most of these files are gathered in public databases like the European Nucleotidic Archive (ENA)^1^ that weighs 54.5 PB by early 2024. The size of the databases follows an exponential growth, and thus we need appropriate solutions to manipulate the data it contains. One simple operation that we are not yet able to achieve (in reasonable time and resources) is to query the database and then, for each dataset, answer if a sequence is present or absent. Even better, answer for each dataset how many times a sequence is present: its abundance. To this end, we use indexing data structures that can handle another representation of the data, making it easier to query afterward. Some of the current indexing data structures use sets of *k*-mers (substrings of length *k*, *k* usually in [20;50]) as the representation to query. In this way, the proportion of shared *k*-mers between a query sequence and a dataset can be determined. The main operation is thus to determine for each *k*-mer in which indexed dataset it occurs and with what abundance (how many times it occurs in a dataset).

Due to the scale of databases to index, state-of-the-art tools often sacrifice precision for the sake of performance. This can be done through pseudo-alignment as defined in Themisto,^2^ breaking down the queried sequences into *k*-mers and comparing them against *k*-mers of the datasets, often encoded as a colored De Bruijn graph, as in Bifrost^3^ or GGCAT.^4^ Here, the graph construction is the main limitation of the methods. Other tools allow false-positive results by using approximate membership queries (AMQ) data structures to enhance space efficiency.^5^^,^^6^^,^^7^^,^^8^^,^^9^^,^^10^ They all have trade-offs between the index size and the false positive rate. By taking advantage of DNA and *k*-mers properties (small alphabet, redundancy of consecutive *k*-mers), the use of a simple associative array with super-*k*-mers^11^ whose minimizers^12^ have been hashed with a minimal perfect hash function^13^ and can create exact and space efficient indexes such as SSHash.^14^^,^^15^ However, apart from being static, exactness requires a trade-off with construction and query times.

Data structures form the core of the tools mentioned above. The choice of the structure impacts the performance and the range of operations available to the user. To illustrate, a Bloom filter^16^ can insert elements after it has been built in memory, while an XOR filter^17^^,^^18^ has better space usage, but is static. A Quotient Filter^19^ allows more dynamicity than a Bloom filter as it can enumerate inserted elements and thus relocate elements in a smaller or larger structure as needed. The Quotient Filter is the backbone of the Counting Quotient Filter (CQF),^20^ which can retrieve not only the presence or absence of a *k*-mer, but also its abundance. However, this structure results in suboptimal index size.

In this paper, we propose a new genomic data indexing structure, an alternative to the CQF called the Backpack Quotient Filter (BQF). It is more space-efficient than the CQF while still offering the same properties (abundance, dynamicity), at the cost of a negligible false-positive rate. We propose a novel way to handle the abundance information. We let the user control the balance between the index size and the precision with which the index encodes the *k*-mer counts/abundances. In addition, we use the fimpera^21^ scheme to reduce each element’s space usage. The BQF supports a large range of operations: random lookup (abundance), insert, enumerate, resize and delete (under circumstances). In total, our tests show that at the price of a false-positive rate below $10^{-5}$%, the BQF can index billions of elements and their abundance, using between 13 and 26 bits per element. Compared to existing solutions, the BQF has the fastest average query time, while being fully dynamic. It is, to our knowledge, the only data structure that cumulates these features.

## Results

We present experimental results on real metagenomic datasets. The objective is to compare the performances obtained with the BQF with those obtained using state-of-the-art data structures for indexing *k*-mers together with their abundances, based on the Quotient Filter: the CQF^20^ and on the counting Bloom Filter: the CBF.^21^ We also included a comparison with Bifrost^3^ and SSHash.^15^ Both of these approaches allow for querying indexed *k*-mers, but they have significant differences in their main features, which are summarized below. Finally, we added a hashtable to the benchmark for a standard comparison.

These results also enable to show the impact of the unique parameter introduced by BQF: the *s* value. We also show the influence of the number of indexed elements on the whole data structure size.

The version used for BQF is v1.0.0. Details about protocols and links to datasets are available online.^22^

### Used datasets

Our results were obtained on three distinct metagenomic datasets in which we exclusively considered *k*-mers present two or more times.

• Dataset “*sea-water34M*”: 34 million Illumina reads from the *Tara* Oceans sequencing project. The uncompressed *fastq* file is 7.7GB. It contains 263M distinct 31-mers and 346M distinct 19-mers occurring at least twice.
• Dataset “*sea-water143M*”: 143 million Illumina reads from the *Tara* Oceans sequencing project. The uncompressed *fastq* file is 33GB. It contains 1.2B distinct 31-mers and 1.5B distinct 19-mers occurring at least twice.
• Dataset “*gut*”: 13 million reads from pig microbiota Pacbio sequencing. The uncompressed *fastq* file is 42GB. It contains 471M distinct 31-mers and 420M distinct 19-mers occurring at least twice.

**Figure 1:**
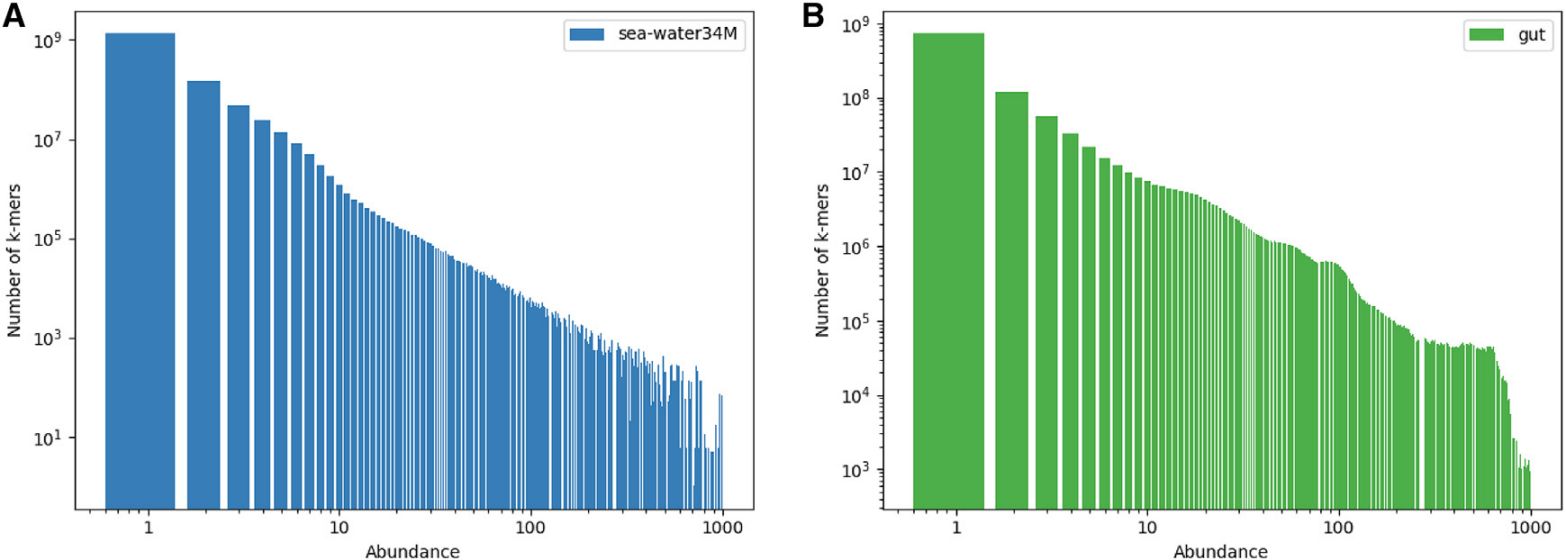
k-mers abundance spectrums for *sea-water34M* and *gut* dataset *k*-mers numbers over (A) *sea-water34M* dataset an (B) *gut* dataset. Abscissa and ordinate are on a logarithmic scale.

These sea-water and gut microbiota metagenomic datasets are representative of a highly complex environment, with a large diversity content. For instance, there are 9.5 billion *k*-mers in *sea-water143M* dataset, leading to a set of 5.7 billion distinct *k*-mers. Among them, only 1.2 billion are present twice or more. For the *gut* dataset, we counted 22 billion *k*-mers, 1.2 billion distinct ones and 0.475 billion are present twice or more. We present in Figure 1 a visualization of the *k*-mer spectrum of the sets *sea-water143M* and *gut*. It shows the complexity of metagenomics dataset. Because of the variety of species sequenced in metagenomics samples, a vast majority of *k*-mers are present once. Additionally, 99% of *k*-mers are present less than 10 times in *sea-water34M* dataset, and 83% for *gut* dataset. Thus, the challenge here is to index low redundant and low abundant *k*-mers.

### Experimental setup

#### Tools

In this section, we present a comparative analysis between BQF and CQF (https://github.com/splatlab/cqf, commit 68939f5). Both structures use the same Xorshift hash function, a PHF, ensuring no collisions. We also compare with results obtained with a counting Bloom filter (CBF), with one hash function, implementing the fimpera approach (https://github.com/lrobidou/fimpera, commit 662328d). Both CBF and BQF use 5 bits for counters (c=5), allowing a maximal abundance value of 64 as we store exact values. BQF and CBF use the fimpera approach, initialized with k=31 and s=19, thus 19-mers are counted and inserted. The sizes of the BQF and CQF are determined solely by the total number of elements plus the element abundances for the CQF. Regarding the CBF, we decided to create a CBF of the same size as the BQF. This ensures fair comparisons when considering a fixed amount of disk space. The choice of parameters is discussed further in this section.

We also show results obtained by Bifrost (version 1.3.1) and SSHash (version 3.0.0). Those comparisons are not exactly fair as these tools embed additional features (computing pre-assembly of the data in the so-called compacted De Bruijn graph, possibly indexing multiple datasets for Bifrost) while Bifrost cannot index the abundance, and while SSHash is a static data structure. However, it is interesting to present these results as they show that these state-of-the-art tools —which are not specifically designed for the task of only indexing *k*-mers with their abundances— are not optimal for this task.

As a standard reference, we also created indexes in the form of hashtables. We used a c++ hashmap available here: https://github.com/martinus/unordered_dense.

#### Parameters and measurements

We computed the sizes on disk, peak memory usage, build time and the query throughput for each approach. In addition to the building time, the results show the pre-processing time, i.e., the time used to obtain the correct input file from the raw compressed *fastq* file (counted *k*-mers for CQF, CBF and BQF, and SPSSs^23^ for SSHash).

The parameters are k=31, c=5 (counters size for BQF, CBF), and s=19 (19-mers were inserted for BQF, CBF). Bifrost used 4 threads and m=17 for SSHash (minimizers size). Finally, the hashtable used 64 bits unsigned integers as keys and 8 bits unsigned integers for abundance values.

Positive queries in a dataset D are *k*-mers reads from D itself. Negative queries are *k*-mers from randomly generated sequences (between 80 and 120 nucleotides). Around 2 billion *k*-mers over 30 million sequences were positively queried. Around 7 billion negative *k*-mers over 100 million sequences were negatively queried.

BQF and CQF sizes are measured experimentally. Their size corresponds exactly to their theoretical value, also showing that, thanks to the simplicity of the structure, no space overhead is required. CBF size was chosen to be the same as BQF’s. SSHash size is the one displayed by the tool at the end of the building step. Bifrost size is measured as the peak memory usage after loading the graph and the index in memory (from binary representation on disk).

The executions were performed on the GenOuest platform on a node with 4×8 cores Xeon E5-2660 2.20 GHz with 128 GB of memory.

### Performance results

#### Comparing CQF and BQF

Compared to the CQF, the major advantage of the BQF is in terms of space. As shown in Table 1, the BQF is approximately four times smaller than the CQF for every indexed dataset. The same advantage is found in terms of space efficiency (bits/element), being approximately 5–7 times more efficient. However, one drawback is the occurrence of false positive calls, which are generally less than $10^{-5}\%$ and can even be as low as 0% in the gut dataset.

**Table 1:** Comparative performances

Dataset	Structure	Index size On disk (GB)	Bits per element	Pre-processing + Build time (s)	Build peak memory usage (GB)	Pos. query throughput (kmer/s)	Neg. query throughput (kmer/s)	FP rate (%)
sea-water34M	Bifrost	5.84	177	1,041α	5.84	2,687,272	3,224,789	0
	SSHash	0.40	12	1,165β + 67	2.46	1,150,224	1,354,394	0
	Hashtable	2.37	71	219γ + 508	8.31	529,901	777,447	0
	CQF	4.58	139	219γ + 210	4.60	1,448,481	2,121,294	0
	CBF	1.11	26	219γ + 429	1.11	205,306	285,061	$4.8\times 10^{-6}$
	**BQF**	1.11	26	219γ + 257	1.11	2,052,016	2,934,776	$1.6\times 10^{-6}$
sea-water143M	Bifrost	17.57	114	6,074α	21.94	1,321,360	2,581,435	0
	SSHash	1.97	13	5,875β + 361	11.15	871,794	1,122,606	0
	Hashtable	11.00	71	780γ + 2563	50.33	316,025	636,648	0
	CQF	17.25	113	780γ + 949	17.52	1,097,099	1,602,930	0
	CBF	3.93	21	780γ + 2,039	3.93	195,177	281,244	$5.8\times 10^{-5}$
	**BQF**	3.93	21	780γ + 1,101	3.93	1,791,640	2,616,583	$3.0\times 10^{-5}$
Gut	Bifrost	5.84	99	5,972α	5.84	8,448,220	3,114,457	0
	SSHash	0.58	10	2,558β + 113	3.79	4,438,401	1,286,876	0
	Hashtable	4.25	71	1,085γ + 941	15.76	872,072	744,324	0
	CQF	8.90	150	1,085γ + 396	9.01	1,598,278	1,948,436	0
	CBF	1.11	21	1,085γ + 468	1.11	352,201	284,545	$1.6\times 10^{-6}$
	**BQF**	1.11	21	1,085γ + 341	1.11	4,582,535	2,821,471	0

Recall that Bifrost and SSHash do not index the same number of elements than CQF, CBF and BQF, explaining the difference in terms of number of bits per element as compared to the structure size. Given its computation time (≥24 hours on the *sea-water143M* dataset), we report SSHash results only for the *sea-water34M* dataset.

α Bifrost does not require pre-processing step.

β BCALM^24^ (https://github.com/GATB/bcalm, version 2.2.3) for unitigs + UST^23^ (https://github.com/jermp/UST, commit b3d0710) for simplitigs.

γ KMC^25^ (https://github.com/refresh-bio/KMC, version 3.2.4) kmer counting.

#### Comparing CBF and BQF

The results presented in Table 1 indicate that the false-positive rate is slightly better with the BQF compared to CBF. However, both approaches still have a very low false positive rate of approximately $10^{-5}$%, which is insignificant for indexing and pseudo-alignment applications. BQF offers several significant benefits over CBF. First, BQF allows for faster time queries, with an average speed improvement of 50 times compared to CBF. Additionally, BQF does not have any theoretical limitations on the number of stored elements, unlike CBF which is designed for a fixed maximum number of elements that cannot be updated. Finally, the elements stored in a BQF (the *s*-mers) can be enumerated, while this is not the case with the CBF.

#### Abundance overestimation due to the fimpera approach

In this work, we did not recompute the so-called overestimation inherent to the fimpera abundance representation. This overestimation is in the order of 1–2% according to the results presented in ^21^, meaning that 1–2% of the abundances of true positive calls are overestimated. Furthermore, for those results that were overestimated, the average difference was shown to be approximately 1.07 times the correct abundance range. All in all, this slight overestimation, limited to less than 2% of the calls, has no significant impact while estimating the abundance of a query composed of at least dozens or hundreds of *k*-mers.

#### Other tools results

As shown by results presented in Table 1, Bifrost is approximately two times slower than BQF to build the data structure and more than twice as slow to perform negative queries. It uses approximately 4.5 times more space per element, and more importantly, it does not provide the abundance of *k*-mers. The SSHash approach, for its part, taking advantage of super-*k*-mers,^11^ uses approximately 2 times less space per element than BQF. However, it is static and is nearly two orders of magnitude slower to construct, drastically limiting its application to large-scale projects. On the other hand, the hashtable exhibits lower space utilization compared to CQF. However, the bottleneck lies in the query process, as no specific optimizations are applied for searching large index. The structure’s generic nature makes it less suitable for handling the scaling quantities of data typical in bioinformatics.

### Impact of size of the indexed *s*-mers

As stated earlier, the BQF structure stores *s*-mers, to emulate *k*-mers at query time, with s≤k. The choice of the *s* value has several consequences that are described in (Section theoretical influence of the s parameter (in STAR Methods)), and that we propose to empirically observe here.

#### Effect of *s* on the number of construction false positives

**Figure 2:**
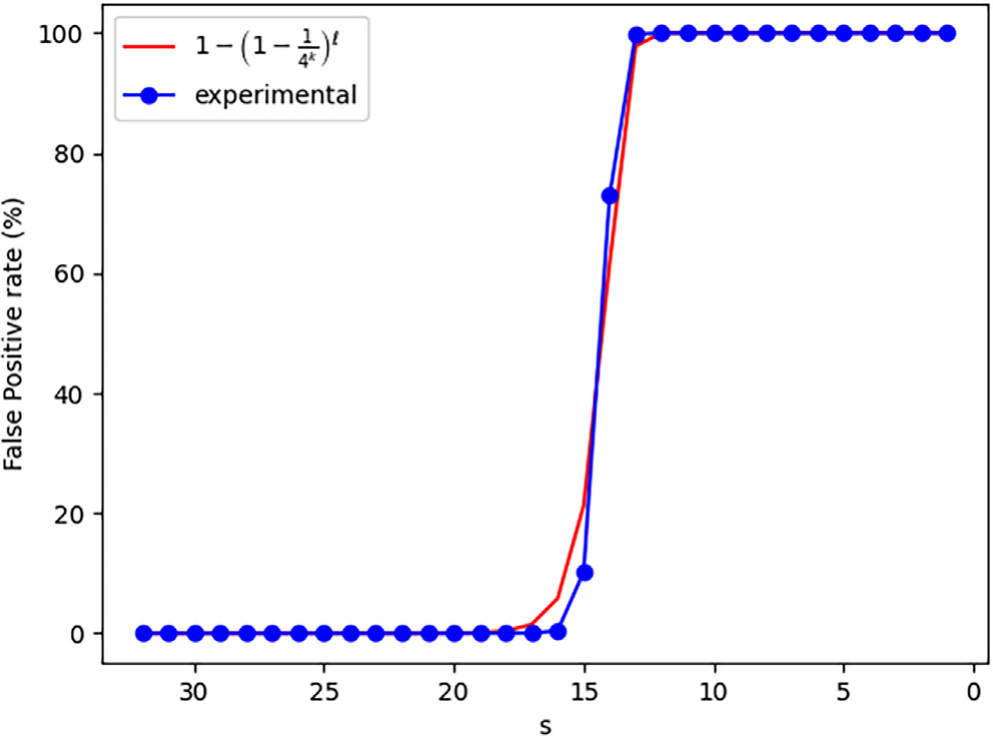
Empirical observation of the evolution of the construction false positive rate with respect to *s* Indexed dataset: “*sea-water34M*”, querying random 31-mers. The value of *s* is decreasing as it starts from *k*, then we increase the difference between *k* and *s*.

Figure 2 illustrates that using any value of *s* bigger than 17 enables to limit drastically the construction false positive rate. When *s*-mers become smaller than 17 nucleotides, the probability they appear by chance in sequences composed of millions of characters on the {A,C,G,T} alphabet becomes close to 1. In this case, mostly any *k*-mers can be constructed from these *s*-mers, explaining the nearly 100% construction false positive rate.

Note that the shape of this curve is highly correlated with the probability that an element of size *s* appears by chance in a sequence of size l. This probability is equal to $1-{\left(1-\frac{1}{4^{s}}\right)}^{l}$. In concrete terms, this allows a user to reliably determine a value of *s* knowing l, even approximately. The value of l can be approximated thanks to the number of distinct *k*-mers in the dataset (as this is the case in Figure 2), efficiently computed by ntCard^26^ for instance.

These results assume a uniform ATCG distribution, we plan for future work to study the impact of high or low GC content.

#### Effect of *s* on the index size

Recall that decreasing *s* has two opposite effects on the structure size.

(a) in certain conditions (see below), decreasing *s* can increase the number of indexed *s*-mers, which tends to increase the size of the structure (need to double its size when reaching 95% load factor);
(b) decreasing *s* decreases the remainder size, and so decreases the total size of the structure.

In this section, we propose to observe the practical consequences of this choice.

**Figure 3:**
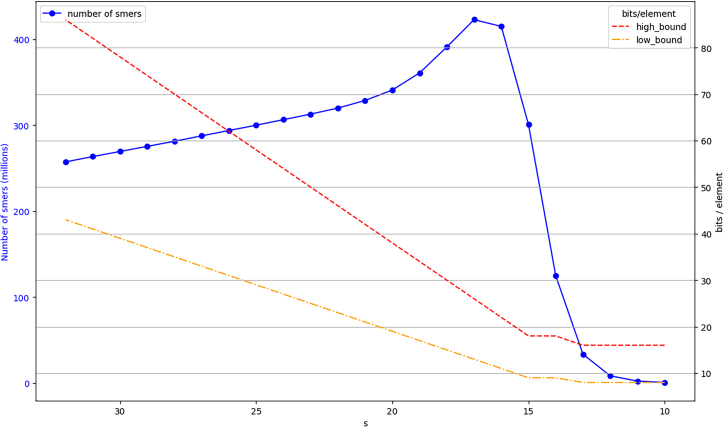
Evolution of the number of *s*-mers depending on *s* in an Illumina sequencing dataset (*sea-water34M*): plain blue line Evolution of the number of bits per element depending on *s* on the same dataset. high_bound is the red upper dotted curve, corresponding to a half-full BQF. low_bound is plotted in orange, under high_bound and corresponds to a full BQF (95% load factor).

(a) Figure 3 shows (plain blue curve) the number of distinct *s*-mers according to *s.* With long enough *s*-mers (s>17), decreasing *s* sub-linearly increases the number of distinct *s*-mers. This is true in the case of relatively short reads, with next generation sequencing for instance (Illumina example within Figure 3 with *sea-water34M* dataset). On the other hand, third-generation sequencing produces longer reads, in this context decreasing *s* decreases the number of elements to index (475M distinct 31-mers and 420M distinct 19-mers in *gut* PacBio dataset). Table 1 shows this result: when comparing BQF and CQF building time (which depends on the number of elements to index), we can see that BQF is slightly faster on *gut* (PacBio) dataset as there are fewer 19-mers than 31-mers.

With s≤17, another effect exists: nearly all the *s*-mers exist in the text, and so the number of distinct *s*-mers becomes limited by $4^{s}$, explaining why the number of distinct *s*-mers decreases when *s* decreases below 7s=1.

(b) The two dashed lines of Figure 3 show the number of bits per element either if the structure is half full or considered as full (in practice one doubles the structure size if its load factor is 95%). The observation is that even on this highly complex sea-water metagenomic dataset, the space needed to store *s*-mers decreases when *s* decreases, even though more *s*-mers have to be stored.

**Figure 4:**
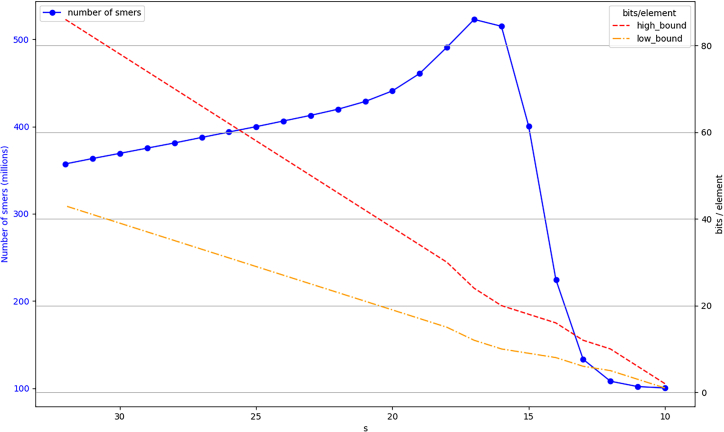
Effect of *s* on the number of *s*-mers in a fictitious dataset (plain blue line) The data has been modified from dataset *sea-water34M* so that the number of *s*-mers reaches a threshold: $2^{29}$ (500M), requiring to double the number of slots. Space efficiency is also plotted (dashed lines) in bits per element with both boundaries: high_bound=50% load factor, low_bound=95% load factor.

When the number of *s*-mers is increasing faster than the number of *k*-mers for the same dataset, there could be the need to double the size of the BQF with *s*-mers requiring $2^{q+1}$ slots, when *k*-mers would have fit in a BQF composed of $2^{q}$ slots. We created a synthetic dataset for generating such a situation, we used *sea-water34M* numbers of *s*-mers for every value of *s* and simply added 100 million elements everywhere. This is for visualization purposes only. Figure 4 shows that, when s=16 or s=17, more than $2^{29}$ elements are to index so $2^{30}$ slots are needed. For these two values of *s*, we can notice that we use even less bits per element (high_bound and low_bound) than for higher *s* values, despite the doubling of the structure size. This is because the more elements we add, the more efficient the structure becomes. This being said, in practice, doubling the size of the structure means that the load factor drops from 95% to 50%, i.e., space efficiency at that moment jumps from low_bound to high_bound. To sum up, in some cases, decreasing *s* might have a momentary negative impact but the overall space efficiency of the BQF continues to improve. It is always beneficial to decrease *s* until we reach the construction false positive threshold, s=17.

All in all, regarding the data-structure size, the best choice is to use *s* as small as possible, but bigger than 17 to avoid an explosion of the construction false positive rate, as it keeps it below $10^{-5}$% in this setup.

### Effect of the number of indexed elements on the structure size

**Figure 5:**
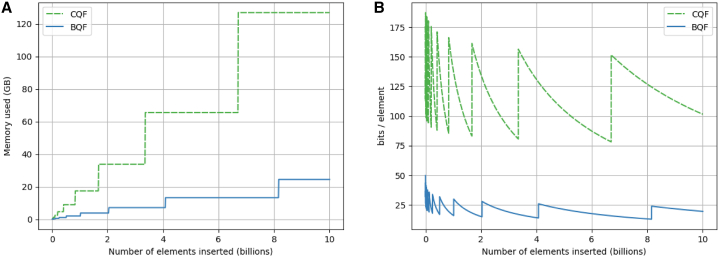
Effect of the number of indexed elements on the size and space efficiency Generated from indexing dataset “*sea-water34M*″, k=31, s=19 and c=5 for BQF. (A) total data-structure size. (B) size in terms of number of bits per indexed element.

Based on our metagenomic samples, this section comments on the experimental value of bits per element (Section number of bits per stored element (in STAR Methods)) used by the BQF compared to CQF. Figure 5 shows the evolution of the data structure size (A) and the evolution of bits per element (B) while elements are inserted.

The stairs shapes of Figure 5A are due to the size of the data structure that doubles each time their load factor reaches 95%. Then, each insertion increases the load factor without consuming more space. The figure highlights the fact that on real metagenomic datasets, the CQF needs a lot of space due to the counter encoding which uses an average of 2.44 slots per element (in the *sea-water34M* dataset). Given a fixed number of insertions, because the CQF doubles its size 2.44 times more frequently, the total size occupied by the CQF is much higher than that of the BQF. At least with metagenomics data, while counts are low but not unique, BQF will always occupy less space than CQF.

In Figure 5B the dented curves show the space used per element. The curves are decreasing as the data structures are filled with elements. The vertical jumps correspond to the data structure resizes. We can see that the two structures behave in the same way while the BQF uses fewer bits per element. It is explained by the number of slots per element (a 2.44 time decrease) but also by the fimpera scheme used in the BQF approach. An interesting fact is that the peaks for both structures get lower while the data structure size doubles. This is because the slots are one bit shorter after each resize, as explained in (Section number of bits per stored element (in STAR Methods)).

Finally, at the price of a negligible non-null false positive rate (in the order of $10^{-5}$% to $10^{-6}$% in our experiments), the BQF enables to make queries among dozens of billions of elements, using between 13 and 26 bits per element, while the CQF requires between 75 and 150 bits per element for the same settings.

## Discussion

This paper introduces the BQF, a quotient filter with abundance. The BQF, like other quotient filters, uses a table to store elements. Only a fraction of these elements is explicitly stored, as the rest is implicitly given through their address. Specifically, in the BQF, for every element, *c* additional bits are used to encode the abundance associated. This strategy enables to index billions of elements with their abundance using between 13 and 26 bits per element, depending on the data structure load factor.

In addition to this counting strategy, the BQF implements the fimpera strategy, which emulates *k*-mers from their *s*-mers (with s≤k). A direct consequence of this emulation is a gain of $2^{q}\times 2\times \left(k-s\right)$ bits over the whole structure, with $2^{q}$ being the number of slots in the BQF. Our results show that the results are robust with respect to the *s* parameter, as long as *s* is bigger than a fixed threshold, namely s>17.

Our results from indexing metagenomic data indicate that the BQF is at least four times more compact than the most similar data structure: the CQF.^20^ The indexing and query times are in the same order of magnitude. This result is at the price of a non-null but extremely low false positive rate ($\approx 10^{-6}$% in our experiment). To fully benefit from the flexible sizes of the counters, if the user can afford it, it is advised to index orders of magnitude (e.g., $log_{2}$ values) instead of exact counts.

The BQF inserts hash values of the elements. By using a perfect hash function, we ensure having no collisions among stored elements. This offers the possibility to enumerate the elements stored in the structure. If the structure gets full when adding elements, this offers a way to relocate all elements after doubling the size of the structure. So, there is no theoretical limit to the number of elements stored in the BQF. This dynamicity is significant in the context of intensive sequencing and indexing.

### Limitations of the study

The main limitation of the BQF is that it is able to index only one source (or sample) of data. One could want to index multiple samples and answer for a query sequence, the set of samples where the sequence is sufficiently present. This is called a colored query and this is a work-in-progress for the BQF. A second limitation is the configuration of the tool. It might be confusing for a new user to understand parameters initialization and sub-optimal performances can occur because of poor choices. Lastly, the BQF do not benefit from data redundancy, which is a good argument for metagenomics data but it might underperform for other data types, pangenomics, for instance.

## Resource availability

### Lead contact

Requests for further information and resources should be directed to and will be fulfilled by the lead contact, Victor Levallois (victor.levallois@inria.fr).

### Materials availability

The materials in this study is available and public. This study did not generate new unique reagents.

### Data and code availability

• Sequences data have been deposited at a data repository and are publicly available as of the date of publication. Accession numbers and links are listed in the key resources table.
• All code is publicly available as of the date of publication. DOIs are listed in the key resources table.
• Any additional information required to reanalyze the data reported in this paper is available from the lead contact upon request.

## Acknowledgments

The work was funded by the Inria Challenge “OmicFinder”, the ANR SeqDigger (ANR-19-CE45-0008), and the European Union’s Horizon 2020 research and innovation program under the Marie Skłodowska-Curie grant agreement No 956229. The funders had no role in study design, data analysis, decision to publish, or manuscript preparation. We acknowledge the GenOuest bioinformatics core facility https://www.genouest.org for providing the computing infrastructure. The authors thank Émeline Roux for providing the link to the *gut* dataset.

## Author contributions

Conceptualization, V.L., F.A., P.P., and Y.D.; methodology - implementation, V.L. and F.A.; investigation, V.L.; writing – original draft, V.L.; writing – review and editing, V.L., P.P., Y.D., F.A., and B.L.G.; funding acquisition, P.P.; supervision, P.P., Y.D., and B.L.G.

## Declaration of interests

The authors declare no competing interests.

## STAR★Methods

### Key resources table

**Table:** 

REAGENT or RESOURCE	SOURCE	IDENTIFIER
**Biological samples**		

Metagenomics sea water Tara Oceans samples	European Nucleotidic Archive (ENA)	ftp://ftp.sra.ebi.ac.uk/vol1/run/ERR172/ERR1726642/AHX_ACXIOSF_6_1_C2FGHACXX.IND4_clean.fastq.gz
Metagenomics sea water Tara Oceans samples	European Nucleotidic Archive (ENA)	ftp://ftp.sra.ebi.ac.uk/vol1/run/ERR599/ERR599283/AHX_ATRIOSF_7_1_C0URMACXX.IND4_clean.fastq.gz
Metagenomics pig gut samples	Holopig project	https://ng6.toulouse.inra.fr/fileadmin/data_seqoccin/analyze/af80b3c73/m64122_220509_072836.hifi_reads.fastq.gz

**Deposited data**		

Sequence datasets	Zenodo record	https://zenodo.org/records/13992590
Metagenomics sea water Tara Oceans samples	European Nucleotidic Archive (ENA)	ERR1726642
Metagenomics sea water Tara Oceans samples	European Nucleotidic Archive (ENA)	ERR599283

**Software and algorithms**		

python 3.9.5	Python	https://www.python.org/
KMC 2.3.0	Kokot et al.^25^	https://doi.org/10.1093/bioinformatics/btx304 https://github.com/refresh-bio/KMC
CQF commit 68939f5	Pandey et al.^20^	https://doi.org/10.1145/3035918.3035963 https://github.com/splatlab/cqf
Bifrost 1.3.5	Holley and Melsted^3^	https://doi.org/10.1186/s13059-020-02135-8 https://github.com/pmelsted/bifrost
Sshash 3.0.0	Pibiri^14^	https://doi.org/10.1093/bioinformatics/btac245 https://github.com/jermp/sshash
CBF commit 662328d	Robidou and Peterlongo^21^	https://doi.org/10.1093/bioinformatics/btad305 https://github.com/lrobidou/fimpera
BQF	This paper	https://github.com/vicLeva/bqf/

### Method details

#### Preliminaries

##### *k*-mers, pseudo-alignment, and indexing

A *k*-mer is any sequence of given size *k*. It can be of any character but in our context a *k*-mer is a substring of a genomic sequence, i.e., made up of nucleotides (A,C,G,T). The number of *k*-mers existing in two sequences provides a metric to measure the similarity between them, leading to the so called pseudo-alignment.^2^ In order to efficiently perform pseudo-alignments between any queried sequence and a dataset, we index its *k*-mers. Doing so, it is possible to know whether a *k*-mer belongs to the dataset or not. Then, when querying a sequence *S*, all of its *k*-mers are individually queried in the index, enabling to compute the pseudo-alignment between *S* and each dataset of a collection.

##### Hash function

A hash function is a mathematical transformation that takes an input (here a sequence of characters) and produces a number, called a hash value. In the current framework, the used hash function produces a value that is coded with a fixed-size number of bits. This transformation is designed to be deterministic, to produce an uniform distribution, and we want it to be as fast as possible.

Given a hash function, two distinct elements are said in “collision” if they have the same hash value. In this paper, we made the choice to use a xorshift^27^ hash function, producing numbers between 0 and $2^{2k}$ for every *k*-mer. We use this^28^ xorshift hash function as it is a injective, preventing collisions.

Also, as long as we project the *k*-mers into values of 2k bits, the function is reversible. It means that we can retrieve the original *k*-mer from its hashed value. The use of a non injective hash function would also be possible but would imply the impossibility to enumerates the elements entered in the data structure. This would prevent, for example, the resizing of the data structure. As we want a fully dynamic data structure, we made the choice of using the injection.

Finally, we need a hash function to randomize the positions of elements in the structure and thus avoid the creation of long runs of elements that would slow down insertions and queries.

##### Quotient filter (QF)

The work we present, called the BQF, is based on the Quotient Filter (QF) structure.^19^ In this section, we provide a brief overview of the fundamental aspects of the QF structure, which is essential for comprehending our contribution.

A QF is a data structure that is used to store a set of elements. It is composed of a table with $2^{q}$ slots, each of fixed size *r*, where *q* and *r* are initially defined by the user. *q* and *r* are subject to change as the size of the table may change. It utilizes a hash function *h* that hashes elements to integers of q+r bits. When an element *x* is inserted, its hash value h(x) is computed and split into two parts:

• $h_{0}\left(x\right)$ of size *q* bits, called the “quotient”. It is used as an address in the table;
• $h_{1}\left(x\right)$ of size *r* bits, called the “remainder”. It is a fingerprint and is effectively stored in memory. $h_{1}\left(x\right)$ is inserted at the address $h_{0}\left(x\right)$.

To query the presence of an element *y* in the structure, $h_{0}\left(y\right)$ and $h_{1}\left(y\right)$ are computed. Finding $h_{1}\left(y\right)$ at position $h_{0}\left(y\right)$ implies that *y* is, with known probability, present. See below pictures the insertion step at slot 3, where solid hatched green lines symbolize the *r* bits of the remainder, inserted at address 3.

**Figure:**
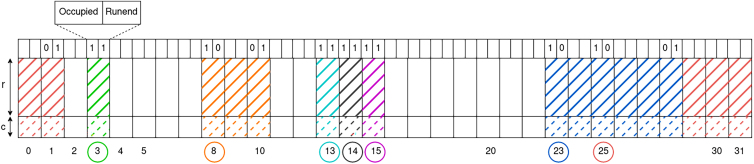
A 32 slots long BQF (*q* = 5) First line represents the metadata bits (see Pandey et al.^20^ for more details). This short example does not represent blocks (Section block-based optimization) in the BQF for simplicity. Each slot has a size of *r* bits for the remainder with *c* bits for counts and 2 bits of metadata: occupied and runend. A circled address *Q* means that at least one element *x* such that $h_{0}\left(x\right)=Q$ has been inserted. Multiple remainders sharing the same color in the BQF have been originally inserted at the same address and form a run. Empty metadata bits are set to 0.

##### Soft collision resolution and run management

We differentiate two kinds of hash collision. One is called “hard collision”, and happens when two distinct elements have the same hash value. We avoid this by using a PHF. The second is called “soft collision”. It is inherent to quotient filters. A soft collision occurs when two distinct elements *x* and *y* have different hashes but the same quotient: $h_{0}\left(x\right)=h_{0}\left(y\right)$.

Because only one remainder can be inserted in any slot, additional remainders sharing the same quotient value are shifted into the next slots. Elements in soft collision are stored consecutively in the table, thus forming a so-called “run”. Inside a run, the remainders are stored in ascending order. Formally, for all elements x,y in a run, with $h_{0}\left(x\right)=h_{0}\left(y\right)$, and assuming that $h_{1}\left(x\right)<h_{1}\left(y\right)$, the slot address where *x* is stored is lower than the one of *y*.

The slot address of the first element $x_{first}$ of a run may be distinct from $h_{0}\left(x_{first}\right)$. The run can be shifted further than its insertion slot. This case appears when another upstream run already occupies the slot given by $h_{0}\left(x_{first}\right)$.

Note that an element shifted from the last slot ($2^{q}-1$) goes into slot 0, since the structure is circular.

To keep track of the shifting process for later insertions and queries, two additional bits of metadata are used in each slot. They enable, thanks to rank and select operations, to determine the actual slot where an element is stored. Finally, an additional bit per slot is used to improve the theoretical complexity of the insertion and query and the practical speed.

• The occupied bit determines whether a slot welcomed an element, whose remainder could be shifted and located elsewhere. In Figure 6, slot 3 has its occupied bit set to one because the green run (1 element) has been inserted here. In slot 25, the occupied bit is also set to 1 because it is the insertion slot of the red run, even though the red run has been shifted further by the blue run.
• The runend bit indicates whether a slot stores a remainder that is at the end of a run. In this case, it is set to 1. Otherwise, it is set to zero, and in this situation, the next slot is either empty or is the first one of a different run.

In Figure 6, 3 remainders are forming a run on slots 8, 9, 10. They all have been inserted with the quotient being 8 and then the biggest ones were shifted to the right. This simple run of 3 elements differs from slots 13, 14, 15 where we have 3 runs of 1 element each.

If we consider a CQF composed of $2^{q}$ slots, then we have two binary vectors: occupieds and runends, both of size $2^{q}$ bits. To find a possibly shifted run from a slot *i*, we aim to find the end of this run. One way to do so is by:

(1) counting the number *d* of runs that are present before *i* by counting the number of 1 in occupieds before occupieds[i]. We call this operation Rank(occupieds,i). Rank(v,i) is defined as the number of 1’s from position 0 to position *i* (included) in a binary vector *v.*
(2) finding the position of the $d^{th}1$ in runends. Here we have the second operation: Select(runends,d), and we define Select(v,i) = position of the $i^{th}$ 1 in the vector *v.*

All in all runend_position(i)=Select(runends,Rank(occupieds,i)).

##### Block-based optimization

When inserting or querying an element in the filter, the position of the run where it belongs needs to be computed. This means applying Rank and Select over occupieds and runends. As it becomes too expensive to iterate over a billion bits long vectors, the structure is divided into blocks of 64 slots. Each block acts as a checkpoint and stores an additional information: Offset, stored on 64 bits. The Offset of the slot *i* is the distance between *i* and the last slot of its run. We store the Offset of the first slot of each block. Because we store an additional 64 bits number every 64 slots, it increases the number of metadata bits per slot to 3. As shown in,^20^ we can encode the Offset so that it uses 0.125 bits per slot instead of 1. In our implementation, we still made the choice to use 64 bits for every Offset, bringing the number of metadata bits to 3 instead of 2.125 for memory alignment reasons. Thanks to the Offset information, it is now possible to count the number *d* of runs that started before the slot *i* and after the first slot (*j*) of the same block. Then we jump to the position given by Offset(j) and we find the $d^{th}$ runend from there.

In summary, we compute runend_position(i)=Select(runends[Offset(j),i−1],d), with d=Rank(occupieds[j,i],i).

Originally the QF is a probabilistic data structure: with a non-zero false-positive rate when querying elements. In the current framework, as we use an injective hash function, the false-positive rate is zero. However, as explained later (Section: The Backpack Quotient Filter; subheading: Reducing the Space Usage), we use an additional technique that does not exactly query the actual stored elements, and that generates a negligible but non-zero false positive rate at query time.

In practice, the metadata bits used, the probing method and the global organization of the QF we use is based on the Rank & Select Quotient Filter first proposed in.^20^

##### Abundance in quotient filters

As previously defined, the Quotient Filter structure is enough to handle the presence or absence of *k*-mers. It is possible to adapt the structure so it can store each *k*-mer alongside with its abundance in the indexed dataset. The Counting Quotient Filter^20^ (CQF) is an example of a QF with abundance.

In the CQF, the abundance of each inserted element can be stored using the following process. A slot is used to store a remainder or an abundance value. If an element *x* has its abundance 1≤n≤2, then the element is inserted *n* times (with n=2, two consecutive slots store $h_{1}\left(x\right)$). When n>2, $h_{1}\left(x\right)$ is stored twice and these two slots act like boundaries in the table, defining the beginning and the end of the counter. Then n−2 is encoded and stored between both boundaries, using potentially two slots or more. An extra slot holding a 0 might be necessary to maintain consistency in the runs. The point here is that this approach uses 2 slots when n=2 and 3 or more when n>2.

In the following section, we present our contribution, called the BQF, improving both the way the counts are stored and highly optimizing the size taken by each element to be queried.

#### The backpack quotient filter

##### Storing the abundance

In the BQF, the abundance of each element is stored using the following approach. As represented in Figure 6 each slot stores both a remainder and an abundance value. More precisely, each slot stores *r* bits for the remainder, and *c* extra bits are used to encode the abundance value. The *c* parameter is a user-defined parameter. The choice of *c* has a direct impact on the BQF size, adding $2^{q}\times c$ bits. The maximum value for abundance is $2^{c}-1$ and the value can be an exact count, or an order of magnitude (e.g., encoding of $log_{2}$ values), offering flexibility based on precision requirements.

If the value of a count overflows the number of bits allocated, the $2^{c}-1$ value is stored and the answer at query time is “$\geq 2^{c}$”.

Compared to the CQF, the BQF uses only one slot per element, regardless of the abundance of this element, but at the same time it uses more bits per slot. In the end, the BQF will use *c* bits to encode the abundance, while the CQF will use x×r bits (*x* slots, *x* usually between 1 and 4). Instinctively, the larger the CQF’s *r* parameter is, the more interesting our method is. But the main point is that *r* is not user-defined but more inferred by the hash and *q*, while *c* can be determined according to available space and counts. For instance, one might just want to know that a count is greater than a certain value ($2^{c}$), to be considered “big” and not worry about whether the number is 10 or 1000 times greater.

On a side note, the name BQF comes from the fact that every slot handles its own counter, as if it was carrying a backpack.

##### Reducing the space usage

In order to reduce the space usage, we take advantage of a method called Fimpera.^21^ This method is originally designed to reduce the false-positives of data-structures having non-zero false positive rates.

Focusing on the presence/absence only, the key idea can be summarized as follows: if a word is present in a text, then all of its sub-words are present. Conversely, if any sub-word is absent, then the whole word is absent. In practice, instead of indexing the *k*-mers from a dataset, we insert all its *s*-mers, with s≤k. At query time a *k*-mer is considered as indexed if and only if all its *s*-mers are indexed in the structure. In the general case of querying a *k*-mer in an structure with collisions, this approach enables to lower the false positive rate of the query because all *s*-mers of a specific *k*-mer need to be false positives to create a false positive *k*-mer.

The same idea can be exploited when taking the abundance into account. The abundance of a *k*-mer is at most equal to the least abundant *s*-mer it is composed of. Therefore, we store the abundance of s-mers in the filter and report the abundance of a queried k-mer as the minimum of the abundances of the s-mers composing it. The techniques described in^21^ explain how this approach does not have a negative impact on query time and may even improve it. When applied to a structure having collisions, this approach limits the overestimation of the abundance, as all the *s*-mers of a queried *k*-mer have to be overestimated to overestimate the real abundance of this *k*-mer.

In the BQF, we do not have any collision. We apply this approach to gain space instead.

Let us first study the size of the reversible hash value, used to store words on a four-character alphabet. Each character (here {A,C,G,T}) requires two bits for its encoding. Hence, encoding a word of length l requires 2l bits. As we use a reversible hash function, the size of the hash value requires the same size as the original encoded data, 2l.

By inserting *s*-mers, smaller than *k*-mers, the size of the reversible hash value of each inserted element becomes 2s instead of 2k. If we denote by *z* the difference between *k* and *s*, the gain is 2z bits per element. In the BQF structure, the consequence is that the size of each slot is decreased by 2z. All in all, applying this approach enables to save $2^{q}\times 2z$ bits. The same hash function is used, with the same properties of injection and reversibility of stored elements.

A drawback of using this approach is the loss of the enumerating feature for *k*-mers. The hash function is still reversible but because we have *s*-mers in the filter, we can only reconstruct (and thus enumerate) these *s*-mers and not the *k*-mers we want to query. It is important to note that we only lose the *k*-mers enumeration, not the dynamicity: resizing the BQF remains possible.

If the counters are not exact, i.e., if orders of magnitude are indexed then inserting and deleting new elements is no longer a trivial task. We will study the possibilities of updating the BQF in this case in future work.

The second drawback of applying this approach is the creation of a new kind of false positives, called “construction false positives”. The existence of construction false positive is explained by a simple sentence: a *k*-mer may be absent but all of its *s*-mers may be present. We meet this case if each *s*-mer of an absent *k*-mer *x* has been individually inserted through the present *k*-mers sharing *s*-mers with *x*. Overestimations can also happen, a study of this probability has been realised in fimpera paper.^21^

##### Theoretical influence of the *s* parameter

We now detail the theoretical consequences of reducing the size *s* of indexed elements, with s∈[0,k].

(1) Decreasing *s* increases the “construction false positive” rate. The smaller the *s* value is, the higher is the probability that a queried *k*-mer, non existing in the indexed set, has all its *s*-mers existing in this set.
(2) Decreasing *s* may increase the number of indexed *s*-mers in short reads datasets. A sequence of size l contains (l−k+1) *k*-mers and (l−s+1) *s*-mers. Hence, it contains z=k−s additional *s*-mers than *k*-mers. This is negligible while indexing for instance an assembled genome. But when it comes to index millions of reads with low redundancy between them, as this is the case in our experimentations using sea-water metagenomes, each of the million reads contains *z* more *s*-mers than *k*-mers, with a low redundancy between reads.
(3) Decreasing *s* decreases the size taken by each indexed *s*-mer, which is the expected effect. This is the main advantage of the approach. Recall that the total size of structure is reduced by $2^{q}\times 2z$ when using *s*-mers instead of *k*-mers. Hence the smaller *s* is, the more space is saved.

In general, the results presented (Section impact of size of the indexed s-mers (in results)) suggest that the size of the data structure decreases as *s* decreases, despite the conflicting effects of the last two previous points. Selecting small *s* values only has the potential to increase the construction false positive rate. However, when using recommended values, it stays below $10^{-5}\%$.

##### Doubling the number of slots when the structure is full

One of the main advantage of building the QF with an injective hash function is that conversely to a Bloom filter for instance, when the structure is full, it is possible to double its number of slots (from *q* to q+1). During this process, the hash value of each element remains the same, but the way it is distributed between the quotient and remainder changes. This occurs because, after doubling, q+1 bits are used to represent the address, while r−1 bits are used for the remainder. Finally, the total number of stored elements faces no theoretical limitation.

In practice, for performances reasons, one doubles the number of slots when the “load factor” (number of stored elements divided by the number of slots) becomes bigger than 95%. Load factor effect experiments have been performed here.^20^

##### Number of bits per stored element

As stated, the basis of the QF data structure is to use the address of stored elements as a part of their hash value. As a consequence, the size of the remainder stored for each element decreases when the number of slots increases. This is not linear. Let us consider the initial scenario, where the QF is composed of $2^{q}$ slots in which *r* bits per slot are used as remainder. In this case, the BQF uses $2^{q}\times \left(r+c+3\right)$ bits, as for each stored element, *r* bits store the remainder, *c* bits are used to store the abundance, and 3 additional metadata bits are used by the structure itself (runend, occupied and Offset).

Now consider that the size of the structure doubles in order to index more elements. The structure then contains $2^{q+1}$ slots. In this situation, q+1 bits indicate the address of each slot, and so the remainder of each element decreases to r−1 bits instead of *r*. In this case, the total size of the structure becomes $2^{q+1}\times \left(r-1+c+3\right)=2^{q+1}\times \left(r+c+2\right)$. As the structure grows, q+r remains constant and the slots become smaller.

Note that this practical effect ends when the remainder is empty, in which case the full hash value of each element is entirely given by the address of the element. This presents a theoretical perspective. In the case of *k*-mer indexing, where conventional *k* values are typically around 30, approximately 140 petabytes would be needed to contain the $4^{k}$ slots (representing the number of possible distinct *k*-mers).

### Additional resources

Availability: https://github.com/vicLeva/bqf.

## References

[1] Burgin J., Ahamed A., Cummins C., Devraj R., Gueye K., Gupta D., Gupta V., Haseeb M., Ihsan M., Ivanov E. (2023). The european nucleotide archive in 2022. Nucleic Acids Res..

[2] Alanko J.N., Vuohtoniemi J., Mäklin T., Puglisi S.J. (2023). Themisto: a scalable colored k-mer index for sensitive pseudoalignment against hundreds of thousands of bacterial genomes. Bioinformatics.

[3] Holley G., Melsted P. (2020). Bifrost: highly parallel construction and indexing of colored and compacted de Bruijn graphs. Genome Biol..

[4] Cracco A., Tomescu A.I. (2023). Extremely fast construction and querying of compacted and colored de Bruijn graphs with GGCAT. Genome Res..

[5] Bradley P., den Bakker H.C., Rocha E.P.C., McVean G., Iqbal Z. (2019). Ultrafast search of all deposited bacterial and viral genomic data. Nat. Biotechnol..

[6] Bingmann T., Bradley P., Gauger F., Iqbal Z., Brisaboa N.R., Puglisi S.J. (2019). *String Processing and Information Retrieval*. Lecture Notes in Computer Science.

[7] Pandey P., Bender M.A., Johnson R., Patro R., Berger B. (2018). Squeakr: an exact and approximate k-mer counting system. Bioinformatics.

[8] Lemane T., Medvedev P., Chikhi R., Peterlongo P. (2022). kmtricks: efficient and flexible construction of Bloom filters for large sequencing data collections. Bioinform. Adv..

[9] Srikakulam S.K., Keller S., Dabbaghie F., Bals R., Kalinina O.V. (2023). MetaProFi: an ultrafast chunked Bloom filter for storing and querying protein and nucleotide sequence data for accurate identification of functionally relevant genetic variants. Bioinformatics.

[10] Marchet C., Limasset A. (2023). Scalable sequence database search using partitioned aggregated Bloom comb trees. Bioinformatics.

[11] Li Y., Kamousi P., Han F., Yang S., Yan X., Suri S. (2013). Memory efficient minimum substring partitioning. Proceedings VLDB Endowment.

[12] Roberts M., Hayes W., Hunt B.R., Mount S.M., Yorke J.A. (2004). Reducing storage requirements for biological sequence comparison. Bioinformatics.

[13] Pibiri G.E., Trani R. (2021). Proceedings of the 44th International ACM SIGIR Conference on Research and Development in Information Retrieval. SIGIR ’21 New York, NY, USA.

[14] Pibiri G.E. (2022). Sparse and skew hashing of K-mers. Bioinformatics.

[15] Pibiri G.E. (2023). On weighted k-mer dictionaries. Algorithms Mol. Biol..

[16] Bloom B.H. (1970). Space/time trade-offs in hash coding with allowable errors. Commun. ACM.

[17] Graf T.M., Lemire D. (2020). Xor Filters: Faster and Smaller Than Bloom and Cuckoo Filters. ACM J. Exper. Alg..

[18] Graf T.M., Lemire D. (2022). Binary Fuse Filters: Fast and Smaller Than Xor Filters. ACM J. Exper. Alg..

[19] Bender M.A., Farach-Colton M., Johnson R., Kraner R., Kuszmaul B.C., Medjedovic D., Montes P., Shetty P., Spillane R.P., Zadok E. (2012). Don’t Thrash: How to Cache Your Hash on Flash. arXiv.

[20] Pandey P., Bender M.A., Johnson R., Patro R. (2017). *Proceedings of the 2017 ACM International Conference on Management of Data*. SIGMOD ’17 New York, NY, USA.

[21] Robidou L., Peterlongo P. (2023). fimpera: drastic improvement of Approximate Membership Query data-structures with counts. Bioinformatics.

[22] Experiments details and protocols. https://github.com/vicLeva/bqf/wiki/Experiments-details-and-protocol-for-BQF-paper-results.

[23] Rahman A., Medvedev P. (2020). Research in Computational Molecular Biology - 24th Annual International Conference, RECOMB 2020, Padua, Italy, May 10-13, 2020, Proceedings vol. 12074 of Lecture Notes in Computer Science.

[24] Chikhi R., Limasset A., Medvedev P. (2016). Compacting de Bruijn graphs from sequencing data quickly and in low memory. Bioinformatics.

[25] Kokot M., Dlugosz M., Deorowicz S. (2017). KMC 3: counting and manipulating k-mer statistics. Bioinformatics.

[26] Mohamadi H., Khan H., Birol I. (2017). ntcard: a streaming algorithm for cardinality estimation in genomics data. Bioinformatics.

[27] Marsaglia G. (2003). Xorshift RNGs. J. Stat. Softw..

[28] Wang T. (1997). Integer Hash Function. https://gist.github.com/lh3/59882d6b96166dfc3d8d.

